# Supplementary material for: Changes in health-related quality of life in young-old and old-old patients undergoing elective orthopedic surgery: A systematic review
Source: PLoS One. 2024 Oct 1;19(10):e0308842. doi: 10.1371/journal.pone.0308842 (PMC11444409; doi:10.1371/journal.pone.0308842)
Supplement: S1 File — (DOCX) [file pone.0308842.s001.docx]

**Supplementary Material**: Changes in health-related quality of life in young-old and old-old patients undergoing elective orthopedic surgery: A systematic review

Table of Contents

[S1 Table. Meta-analysis of Observational Studies in Epidemiology assessment 2](#_Toc159075359)

[S2 Table. Newcastle-Ottawa scale assessment 5](#_Toc159075360)

[S3 Table. Absolute change in EQ-5D values in hip and knee arthroplasty patients. 7](#_Toc159075361)

[S4 Table. Absolute change in SF-36 or SF-12 values in hip and knee arthroplasty patients. 8](#_Toc159075362)

[S5 Appendix: PRISMA Checklist 9](#_Toc159075363)

[S6 Appendix: MEDLINE search strategy 11](#_Toc159075364)

Supplemental Table 1: Meta-analysis of Observational Studies in Epidemiology assessment

| First author, year | Study design | Study population clearly identified? | Clear definition of outcome and outcome assessment? | | Selective loss of patients during the follow up? | | Important confounders and / or prognostic factors identified | NOS scores |
| --- | --- | --- | --- | --- | --- | --- | --- | --- |
| Aalund, 2017, (18) | PC | yes | yes | no | | Multiple linear regressions used for analysis. Analyses regarding impact of preoperative HRQoL were adjusted for sex and age. The minimum clinically relevant difference was set at 0.03. | | 6 |
| Anderson, 2022, (20) | PC | yes | yes | no | | Age, gender, comorbidities, preoperative level of hemoglobin and glomerular filtration rate, surgery, ASA score, hospital length of stay. Wilcoxon test used for paired samples.  In case of significant results, Dunn ’ s method was used for the following pairwise comparison. Categorical data were presented as rates and intergroup comparisons was performed by the Chi-Square test. | | 5 |
| Clement, 2022, (46) | PC | yes | yes | no | | t - test, paired and independent- samples, and ANOVA or Kruskal Wallis, with post hoc Bonferroni correction for multiple testing, were used to compare linear variables between groups. Dichotomous variables were assessed using a chi-squared or fisher's exact test. | | 6 |
| Gordon, 2014, (21) | PC | yes | yes | no | | Sex, patient-reported Charnley class, previous contralateral THR (total hip replacement), and preoperative pain. Linear regression model was used to adjust for the listed confounders | | 8 |
| Miao,  2018, (22) | PC | yes | yes | NR | | Age, sex, occupation, place of residence, surgery type, comorbidities, postoperative complications, home environment. ANOVA was conducted for the EQ-5D, satisfaction, and knee and hip functioning data. A multivariable analysis was conducted to determine factors associated with the HRQoL over time. | | 7 |
| Tadros, 2018, (40) | PC | yes | yes | no | | Age, sex, BMI, surgery, ASA. ANOVA and post hoc tests were used to analyse knee scores amongst the groups. Odds ratio was used to compare the risk of revision between the groups. | | 7 |
| Williams, 2013, (23) | PC | yes | yes | no | | Age, gender, implant type (UKR or TKR), BMI at surgery and ASA.  Linear, logistic and ordinal regression modelling were used to describe the association between age and outcomes for continuous, binary and ordinal outcomes, respectively. A likelihood ratio test was used for evidence of non-linear trends, comparing a model with age as a categorical variable to a model with age as an ordinal variable. | | 8 |
| Alentorn-Geli a, 2013, (19) | PC | yes | yes | NR | | Age, gender, surgery side, BMI. Chi-square analysis for categorical and Mann-Whitney U test for quantitative variables were used. | | 6 |
| Ayers, 2022, (44) | PC | yes | yes | no | | Multivariate regression analysis with 95% confidence interval (CI) was performed to determine if age is a significant determinant. The independent variables used in the regression model: age, gender, race and ethnicity, BMI associated comorbidity using CCI, marital status, smoking status, education level, previous surgery, number of other painful hip and knee joints, revision, and PROMs, including preoperative ODI, HOOS pain and ADL scores, and SF-36 MCS and PCS scores. | | 7 |
| Jones ,  2001, (41) | PC | yes | yes | no | | Information regarding pain, function, health-related quality of life, sociodemographic characteristics, and medical status was gathered | | 8 |
| Goh,  2020, (43) | PCC | yes | yes | no | | Age, gender, BMI, diagnosis, range of motion. The student’s t-test and chi-squared test were used to analyse continuous and categorical data. | | 4 |
| Goh,  2021, (42) | PCC | yes | yes | no | | Propensity scores generated using logistic regression were used to adjust for confounding variables including gender, BMI, CCI, baseline KOOS-JR, and SF-12 physical and mental scores. Independent sample t-test and chi-squared test were used to analyse continuous and categorical data. | | 5 |

Abbreviations: ADL: Activities of Daily Living; ANOVA: Analysis of Variance; ASA: American Society of Anesthesiologists; BMI: Body mass index; CCI: Charlson Comorbidity Index; HRQoL: Health-Related Quality of Life; HOOS: Hip disability and Osteoarthritis Outcome Score; KOOS-JR, Knee Injury and Osteoarthritis Outcome Score for Joint Replacement; MCS: Mental Component Summary; NOS: Newcastle-Ottawa scale; NR: Not Reported; ODI: Oswestry Disability Index; PCS: Physical Component Summary; PC: Prospective cohort; PCC: Prospective Case-control study; PROM: Patient Related Outcome Measure; SF-36: 36-Item Short Form Health Survey questionnaire; SF-12: 12-Item Short Form health survey; UKR: unicompartmental knee replacement, TKR: total knee replacement

Supplemental Table 2: Newcastle-Ottawa scale assessment

**Prospective cohort studies (PC)**

| Study | Represent-ativeness of the exposed cohort (1) | | Selection of the non-exposed cohort (1) | | Ascertain-ment of exposure (1) | Demonstration that outcome of interest was not present at start of study (1) | Comparability of cohorts on the basis of the design or analysis (1) | Assessment of outcome  (1) | Was follow up long enough for outcomes to occur (1) | Adequacy of follow up of cohorts (1) | Total  (out of 8) |
| --- | --- | --- | --- | --- | --- | --- | --- | --- | --- | --- | --- |
| Aalund, 2017, (18) | | 1 | | 1 | 0 | 1 | 1 | 0 | 1 | 1 | 6 |
| Anderson, 2022, (20) | | 1 | | 1 | 1 | 1 | 0 | 0 | 1 | 0 | 5 |
| Clement, 2022, (46) | | 1 | | 1 | 0 | 1 | 1 | 0 | 1 | 1 | 6 |
| Gordon, 2014, (21) | | 1 | | 1 | 1 | 1 | 2 | 0 | 1 | 1 | 8 |
| Miao,  2018, (22) | | 1 | | 1 | 1 | 1 | 2 | 0 | 1 | 0 | 7 |
| Tadros, 2018, (40) | | 1 | | 1 | 1 | 1 | 1 | 0 | 1 | 1 | 7 |
| Williams, 2013, (23) | | 1 | | 1 | 1 | 1 | 2 | 0 | 1 | 1 | 8 |
| Alentorn-Geli a, 2013, (19) | | 1 | | 1 | 0 | 1 | 2 | 0 | 1 | 0 | 6 |
| Ayers, 2022, (44) | | 1 | | 1 | 0 | 1 | 2 | 0 | 1 | 1 | 7 |
| Jones ,  2001, (41) | | 1 | | 1 | 1 | 1 | 2 | 0 | 1 | 1 | 8 |

**Prospective Case-control study (PCC)**

| Study | Is the case definition adequate? (1) | Represent-ativeness of the cases  (1) | Selection of Controls  (1) | Definition of Controls  (1) | Comparability of cases and controls based on the design or Analysis (2) | | Ascertainment of exposure  (1) | Same method of ascertainment for cases and controls  (1) | Non-response report (1) | Total  (Out of 9) |
| --- | --- | --- | --- | --- | --- | --- | --- | --- | --- | --- |
| Goh, 2020, (43) | 0 | 1 | 0 | 0 | | 2 | 0 | 1 | 0 | 4 |
| Goh,  2021, (42) | 0 | 1 | 0 | 0 | | 2 | 1 | 1 | 0 | 5 |

Supplemental Table 3: Absolute change in EQ-5D values in hip and knee arthroplasty patients.

| First author, year | Age group (years) | Change in EQ-5D scores: mean ± SD | | | Differences in HRQoL change between age groups |
| --- | --- | --- | --- | --- | --- |
|  |  | 3-month | 12-month | 24-month |  |
| Aalund,  2017, (18) | 70-79  >79 | 0.26 ± 0.24  0.29 ± 0.27 | 0.27 ± 0.24  0.30 ± 0.27 | NR | 3-m (P<0.001)  12-m (P<0.001) |
| Anderson,  2022, (20) | 70-79  ≥80 | NR | 0.46 ± 0.30  0.32 ± 0.38 | NR | n.s. |
| Clement,  2022, (46) | 65-74  ≥75 | 0.50 ± 0.20  0.17 ± 0.26 | 0.52 ± 0.22  0.33 ± 0.32 | 0.34 ± 0.18  0.14 ± 0.25 | NR – young-old show more improvement. Unsure if significant. |
| Gordon,  2014, (21) | 71-80  >81 | NR | 0.14 ± 0.18  0.14 ± 0.19 | NR | NR – Similar improvement |
| Miao,  2018, (22) | 66-75  >75 | 0.52 ± 0.46  0.36 ± 0.50 | NR | NR | NR – younger-old show more improvement. Unsure if significant |
| Tadros,  2018, (40) | 70-79  80-89 | NR | 0.3  0.3 | 0.3  0.3 | NR – Similar improvement |
| Williams,  2013, (23) | 65-74  ≥75 | NR | NR | 0.33 ± 0.42  0.28 ± 0.40 | n.s. |

Abbreviations: TKA: Total Knee Arthroplasty; THA: Total Hip Arthroplasty; NR: Not Reported; n.s.: Not Significant; HRQoL: Health-Related Quality of Life; EQ-5D: EuroQol- 5 Dimension.

Supplemental Table 4: Absolute change in SF-36 or SF-12 values in hip and knee arthroplasty patients.

| First author, year | Age group (years) | | Absolute change in MCS scores: mean ± SD | | | | | Absolute change in PCS scores: mean ± SD | | | | | | Differences in HRQoL change between age groups | |
| --- | --- | --- | --- | --- | --- | --- | --- | --- | --- | --- | --- | --- | --- | --- | --- |
|  |  |  | 6-month | 12-month | | 24-month | | 6-month | | 12-month | | 24-month | |  |  |
| SF-36 | | | | | | | | | | | | | | | |
| Alentorn-Geli^a^,  2013, (19) | | <80  ≥80 | NR | 1.3 ± 19.8  -1.3 ± 19.1 | NR | | NR | | 12.1 ± 11.4  9.1 ± 11.4 | | NR | | PCS: n.s.  MCS: n.s. | |  |
| Ayers, 2022, (44) | | 65-74  ≥75 | NR | 2.9 ± 14.7  2.8 ± 15.3 | NR | | NR | | 13.9 ± 13.1  11.1 ± 13.2 | | NR | | NR – Similar MCS improvement. Young-old has slightly more PCS improvement. Unsure if significant. | |  |
| Goh,  2020, (43) | | 65-74  ≥80 | 1.7 ± 14.6  3.1 ± 14.3 | NR | 3.1 ± 14.5  2.8 ± 14.5 | | 15.7 ± 15.5  12.8 ± 15.0 | | NR | | 18.6 ± 14.5  15.7 ± 14.9 | | NR – Similar MCS improvement. Young-old has slightly more PCS improvement. Unsure if significant. | |  |
| Jones^a^, 2001, (41) | | <80  ≥80 | THA:  0.0 ± 14.1  1.0 ± 19.8  TKA:  3.0 ± 15.6  0.0 ± 15.6 | NR | NR | | THA:  12.0 ± 12.5  13.0 ± 12.1  TKA:  9.0 ± 12.8  7.0 ± 12.2 | | NR | | NR | | NR – Young-old has slightly more PCS improvement. | |  |
| SF-12 | | | | | | | | | | | | | | | |
| Goh,  2021, (42) | 65-74  ≥80 | | NR | NR | | -1.2 ± 11.5  0.3 ± 17.2 | | NR | | NR | | 9.5 ± 12.7  5.0 ± 17.1 | | n.s. in both PCS and MCS | |

Abbreviations: TKA: Total Knee Arthroplasty; THA: Total Hip Arthroplasty; MCS: Mental Component Summary; PCS: Physical Component Summary; NR: Not Reported; n.s.: Not Significant; SF-36: 36-Item Short Form Survey; SF-12: 12-Item Short Form Survey; HRQoL: Health-Related Quality of Life.

^a^ likely includes patients <65.

# S5 Appendix: PRISMA Checklist

| **Section and Topic** | **Item #** | **Checklist item** | **Location where item is reported** |
| --- | --- | --- | --- |
| **TITLE** | | |  |
| Title | 1 | Identify the report as a systematic review. | Line 1 |
| **ABSTRACT** | | |  |
| Abstract | 2 | See the PRISMA 2020 for Abstracts checklist. | Lines 25-52 |
| **INTRODUCTION** | | |  |
| Rationale | 3 | Describe the rationale for the review in the context of existing knowledge. | Lines 57-81 |
| Objectives | 4 | Provide an explicit statement of the objective(s) or question(s) the review addresses. | Lines 82-85 |
| **METHODS** | | |  |
| Eligibility criteria | 5 | Specify the inclusion and exclusion criteria for the review and how studies were grouped for the syntheses. | Lines 115-120 |
| Information sources | 6 | Specify all databases, registers, websites, organisations, reference lists and other sources searched or consulted to identify studies. Specify the date when each source was last searched or consulted. | Lines 96-107 |
| Search strategy | 7 | Present the full search strategies for all databases, registers and websites, including any filters and limits used. | Supplementary Table 6 |
| Selection process | 8 | Specify the methods used to decide whether a study met the inclusion criteria of the review, including how many reviewers screened each record and each report retrieved, whether they worked independently, and if applicable, details of automation tools used in the process. | Lines 111-115 |
| Data collection process | 9 | Specify the methods used to collect data from reports, including how many reviewers collected data from each report, whether they worked independently, any processes for obtaining or confirming data from study investigators, and if applicable, details of automation tools used in the process. | Lines 123-124 |
| Data items | 10a | List and define all outcomes for which data were sought. Specify whether all results that were compatible with each outcome domain in each study were sought (e.g. for all measures, time points, analyses), and if not, the methods used to decide which results to collect. | Lines 124-128 |
|  | 10b | List and define all other variables for which data were sought (e.g. participant and intervention characteristics, funding sources). Describe any assumptions made about any missing or unclear information. | Lines 128-129 |
| Study risk of bias assessment | 11 | Specify the methods used to assess risk of bias in the included studies, including details of the tool(s) used, how many reviewers assessed each study and whether they worked independently, and if applicable, details of automation tools used in the process. | Lines 132-142 |
| Effect measures | 12 | Specify for each outcome the effect measure(s) (e.g. risk ratio, mean difference) used in the synthesis or presentation of results. | N/A |
| Synthesis methods | 13a | Describe the processes used to decide which studies were eligible for each synthesis (e.g. tabulating the study intervention characteristics and comparing against the planned groups for each synthesis (item #5)). | Lines 176-184 |
|  | 13b | Describe any methods required to prepare the data for presentation or synthesis, such as handling of missing summary statistics, or data conversions. | Lines 176-184 |
|  | 13c | Describe any methods used to tabulate or visually display results of individual studies and syntheses. | Lines 176-184 |
|  | 13d | Describe any methods used to synthesize results and provide a rationale for the choice(s). If meta-analysis was performed, describe the model(s), method(s) to identify the presence and extent of statistical heterogeneity, and software package(s) used. | Lines 176-184 |
|  | 13e | Describe any methods used to explore possible causes of heterogeneity among study results (e.g. subgroup analysis, meta-regression). | N/A |
|  | 13f | Describe any sensitivity analyses conducted to assess robustness of the synthesized results. | N/A |
| Reporting bias assessment | 14 | Describe any methods used to assess risk of bias due to missing results in a synthesis (arising from reporting biases). | Lines 132-142 |
| Certainty assessment | 15 | Describe any methods used to assess certainty (or confidence) in the body of evidence for an outcome. | N/A |
| **RESULTS** | | |  |
| Study selection | 16a | Describe the results of the search and selection process, from the number of records identified in the search to the number of studies included in the review, ideally using a flow diagram. | Figure 1 |
|  | 16b | Cite studies that might appear to meet the inclusion criteria, but which were excluded, and explain why they were excluded. | Figure 1 |
| Study characteristics | 17 | Cite each included study and present its characteristics. | Table 2 |
| Risk of bias in studies | 18 | Present assessments of risk of bias for each included study. | Lines 208-218 |
| Results of individual studies | 19 | For all outcomes, present, for each study: (a) summary statistics for each group (where appropriate) and (b) an effect estimate and its precision (e.g. confidence/credible interval), ideally using structured tables or plots. | Table 3-6 |
| Results of syntheses | 20a | For each synthesis, briefly summarise the characteristics and risk of bias among contributing studies. | Lines 208-218 |
|  | 20b | Present results of all statistical syntheses conducted. If meta-analysis was done, present for each the summary estimate and its precision (e.g. confidence/credible interval) and measures of statistical heterogeneity. If comparing groups, describe the direction of the effect. | Lines 222-375 |
|  | 20c | Present results of all investigations of possible causes of heterogeneity among study results. | Lines 247-375 |
|  | 20d | Present results of all sensitivity analyses conducted to assess the robustness of the synthesized results. | N/A |
| Reporting biases | 21 | Present assessments of risk of bias due to missing results (arising from reporting biases) for each synthesis assessed. | Lines 208-218 |
| Certainty of evidence | 22 | Present assessments of certainty (or confidence) in the body of evidence for each outcome assessed. | N/A |
| **DISCUSSION** | | |  |
| Discussion | 23a | Provide a general interpretation of the results in the context of other evidence. | Lines 378-452 |
|  | 23b | Discuss any limitations of the evidence included in the review. | Lines 455-459 |
|  | 23c | Discuss any limitations of the review processes used. | Lines 455-459 |
|  | 23d | Discuss implications of the results for practice, policy, and future research. | Lines 462-466 |
| **OTHER INFORMATION** | | |  |
| Registration and protocol | 24a | Provide registration information for the review, including register name and registration number, or state that the review was not registered. | Lines 91-93 |
|  | 24b | Indicate where the review protocol can be accessed, or state that a protocol was not prepared. | N/A |
|  | 24c | Describe and explain any amendments to information provided at registration or in the protocol. | N/A |
| Support | 25 | Describe sources of financial or non-financial support for the review, and the role of the funders or sponsors in the review. | Line 483 |
| Competing interests | 26 | Declare any competing interests of review authors. | Lines 480-482 |
| Availability of data, code and other materials | 27 | Report which of the following are publicly available and where they can be found: template data collection forms; data extracted from included studies; data used for all analyses; analytic code; any other materials used in the review. | N/A |

| 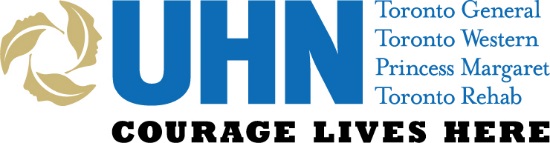S6 Appendix: MEDLINE search strategy | UHN Health Sciences Libraries |
| --- | --- |
|  |  |
|  | **Literature Search Results** |

| **#202313; KS Sys Rev: 3 Selected Health Related Quality of Life Qs in Older Patients with Knee or Hip Surgery (2683)** |
| --- |

For: Dr Frances Chung, Carrie Yun Jin Chen, Yasmin Alhamdah, & Justine Lau

Department: Anesthesia TWH

Date Completed: Monday, May 15, 2023

**Following are your systematic review searches for**:

REVISED SEARCH TOPIC: Hip or Knee Surgery and 3 specific HRQoL surveys and Elderly, limited to English, human, adults. Conference and/or non-journal material removed where/when possible.

**The three selected surveys are:**

Ware, J. E., & Sherbourne, C. D. (1992). **The MOS 36-Item Short-Form Health Survey (SF-36): I. Conceptual Framework and Item Selection**. Medical Care, 30(6), 473–483. PMID: 1593914. <http://www.jstor.org/stable/3765916>

Ware, J. E., Kosinski, M., & Keller, S. D. (1996). **A 12-Item Short-Form Health Survey: Construction of Scales and Preliminary Tests of Reliability and Validity.** Medical Care, 34(3), 220–233. PMID: 8628042. <http://www.jstor.org/stable/3766749>

(1990). **EuroQol - a new facility for the measurement of health-related quality of life.** Health Policy, 16 (3), 199-208. PMID: 10109801.

<https://doi.org/10.1016/0168-8510(90)90421-9>.

**The databases searched were**:

1. [MEDLINE](#Medline); 2. [MEDLINE In-Process/ePubs](#Medline_In_Process); 3. [Embase](#Embase); 4. [CCTR](#CCTR); 5. [CDSR](#CDSR); 6. [Scopus – Citing with Topics Added](#Scopus_Citing_Searching)

**RESULTS & STRATEGY USED**: see following

**Search completed by:** Marina Englesakis, Information Specialist. You may contact me via e-mail at [marina.englesakis@uhn.ca](mailto:marina.englesakis@uhn.ca).

It is important that you are satisfied with your search results.

If you have any questions regarding this search, or if the results were not satisfactory, please do not hesitate in contacting me.

Search strategy saved as: 2023-05-05 FC - Hip or Knee Surgery and 3 HRQoLs and Elderly - Searches

NOTES: preliminary MEDLINE All search strategy approved Monday, May 15, 2023. Searches converted Monday, May 15, 2023.

**Search History**

| **Databases (Platforms)** | **Database Dates covered** | **Date Database  was searched** | **# Citations** | **Notes/Comments** |
| --- | --- | --- | --- | --- |
| MEDLINE (Ovid) | 1946 – May 12, 2023 | May 15, 2023 | 2404 |  |
| MEDLINE ePub Ahead of Print / MEDLINE In-Process & Other Non-Indexed Citations (Ovid) | 2023 May 12 | May 15, 2023 | 158 |  |
| Embase Classic+Embase(Ovid) | 1947 – May 12, 2023 | May 15, 2023 | 3032 | Conference &/or non-journal materials removed at source |
| Cochrane Central Register of Controlled Trials (Ovid) | 1991 – April 2023 | May 15, 2023 | 513 | Conference &/or non-journal materials removed at source |
| Cochrane Database of Systematic Reviews (Ovid) | 2005 – May 9, 2023 | May 15, 2023 | 8 | Full systematic reviews only |
| Scopus (Elsevier) – citation searching of three selected surveys + topics | 1960 – present | May 15, 2023 | 6114 | Journal materials only |
|  |  | Totals: | 12229 | Results in RIS formatted file(s) |
|  |  |  |  |  |

# MEDLINE

Ovid MEDLINE(R) 1946 to May 12, 2023

| **#** | **Searches** | **Results** |
| --- | --- | --- |
| 1 | exp knee arthroplasty/ or total knee replacement/ | 31249 |
| 2 | (knee? adj3 arthroplast*).mp. | 36441 |
| 3 | (knee? adj3 replac*).mp. | 34987 |
| 4 | exp knee prosthesis/ | 13544 |
| 5 | exp knee/ and exp joint prosthesis/ | 674 |
| 6 | (knee? adj3 prosthe*).mp. | 15192 |
| 7 | (knee? adj3 implant*).mp. | 1944 |
| 8 | Arthroplasty, Replacement, Knee/ | 31249 |
| 9 | Knee Prosthesis/ | 13544 |
| 10 | ((knee or knees) adj3 replac*).mp. | 34987 |
| 11 | ((knee or knees) adj3 arthroplast*).mp. | 36441 |
| 12 | ((knee or knees) adj3 prosthe*).mp. | 15192 |
| 13 | (total adj2 knee?).mp. | 27738 |
| 14 | tka.tw. [Total Knee Arthroplasty] | 12875 |
| 15 | tkr.tw. [Total Knee Replacement] | 2030 |
| 16 | tkas.tw. | 2515 |
| 17 | tkrs.tw. | 347 |
| 18 | or/1-17 | 44475 |
| 19 | Knee/ | 16006 |
| 20 | exp Knee Joint/ | 71522 |
| 21 | exp Knee Injuries/ | 29922 |
| 22 | Osteoarthritis, Knee/ | 26825 |
| 23 | Patella/in [Injuries] | 2246 |
| 24 | Patella/su [Surgery] | 3409 |
| 25 | knee.mp. | 172808 |
| 26 | knees.mp. | 24504 |
| 27 | or/19-26 | 183236 |
| 28 | exp Arthroplasty/ | 87398 |
| 29 | exp Joint Injury/ | 7635 |
| 30 | exp Joint Prosthesis/ | 48783 |
| 31 | exp Joints/su [Surgery] | 83299 |
| 32 | exp Leg/su | 5286 |
| 33 | exp Orthopedic Procedures/ | 357389 |
| 34 | Osteoporotic Fractures/ | 7906 |
| 35 | "Prostheses and Implants"/ | 49511 |
| 36 | exp Prosthesis Failure/ | 31739 |
| 37 | arthroplast*.mp. | 94453 |
| 38 | hemiarthroplast*.mp. | 3473 |
| 39 | art#ficial*.mp. | 393051 |
| 40 | hemi-arthroplast*.mp. | 159 |
| 41 | prosthe*.mp. | 332092 |
| 42 | redo.mp. | 5091 |
| 43 | re-do.mp. | 621 |
| 44 | replac*.mp. | 493937 |
| 45 | repair*.mp. | 374230 |
| 46 | revision?.mp. | 101423 |
| 47 | re-revision?.mp. | 532 |
| 48 | rerevision?.mp. | 161 |
| 49 | implant*.mp. | 532578 |
| 50 | endoprosthe*.mp. | 7132 |
| 51 | (surgery or surgeries or surgical* or operation? or operative??).tw,kw. | 2211411 |
| 52 | or/28-51 | 3729828 |
| 53 | 27 and 52 | 102611 |
| 54 | 18 or 53 [Knee Surgery & Related Terms] | 103137 |
| 55 | Arthroplasty, Replacement, Hip/ | 34075 |
| 56 | Hip Prosthesis/ | 25462 |
| 57 | (exp Hip/ or exp Hip Dislocation/ or exp Hip Dislocation, Congenital/ or exp Hip Fractures/ or exp Hip Injuries/ or exp Hip Joint/ or exp Osteoarthritis, Hip/) and exp joint prosthesis/ | 11363 |
| 58 | (exp Hip/ or exp Hip Dislocation/ or exp Hip Dislocation, Congenital/ or exp Hip Fractures/ or exp Hip Injuries/ or exp Hip Joint/ or exp Osteoarthritis, Hip/) and su.fs. | 38677 |
| 59 | (exp Femur Head/ or exp Femur Head Necrosis/) and exp joint prosthesis/ | 2289 |
| 60 | (exp Femur Head/ or exp Femur Head Necrosis/) and su.fs. | 7471 |
| 61 | exp Hip Fractures/ | 28598 |
| 62 | acetabular*.mp. | 16532 |
| 63 | ((femur or femoral) adj3 neck*).mp. | 27655 |
| 64 | ((femur or femoral) adj3 fractur*).mp. | 34124 |
| 65 | femoral head prosthe*.mp. | 125 |
| 66 | femur head prosthe*.mp. | 18 |
| 67 | femoroacetabul*.mp. | 2715 |
| 68 | (hip? adj3 prosthe*).mp. | 27331 |
| 69 | (hip? adj3 arthroplast*).mp. | 41914 |
| 70 | (hip? adj3 replac*).mp. | 40975 |
| 71 | (hip? adj3 implant*).mp. | 2583 |
| 72 | intertrochanteric*.mp. | 3409 |
| 73 | 52 and (exp Hip/ or exp Hip Dislocation/ or exp Hip Dislocation, Congenital/ or exp Hip Fractures/ or exp Hip Injuries/ or exp Hip Joint/ or exp Osteoarthritis, Hip/) | 48324 |
| 74 | (total adj2 hip?).mp. | 36439 |
| 75 | trochanter*.mp. | 8771 |
| 76 | (tha and hip).tw. [Total Hip Arthroplasty] | 10051 |
| 77 | (thr and hip).tw. [Total Hip Replacement] | 2078 |
| 78 | (uka and hip).tw. [Unicompartmental Hip Replacement] | 85 |
| 79 | or/55-78 [ Hip Replacement & related terms ] | 137525 |
| 80 | 54 or 79 [ Knee or Hip Fracture, Injury, Surgery ] | 223826 |
| 81 | "12 item Short Form*".mp. | 1509 |
| 82 | "12-Item* Short Form*".mp. | 1513 |
| 83 | EQ-5D*.mp. | 10232 |
| 84 | EQ5D*.mp. | 788 |
| 85 | EQ5D*.mp. | 788 |
| 86 | EQ5D5*.mp. | 23 |
| 87 | EuroQoL*.mp. | 6736 |
| 88 | EuroQol-5*.mp. | 3427 |
| 89 | EuroQol5*.mp. | 35 |
| 90 | (Europ* adj2 (qol or quality of life) adj2 5*).mp. | 529 |
| 91 | (Europ* adj2 (qol or quality of life) adj2 (index* or instrument* or measur* or questionnaire* or scale* or survey*)).mp. | 355 |
| 92 | (European Quality of Life* adj2 Dimension*).mp. | 532 |
| 93 | European Quality of Life* Five* Dimension*.mp. | 98 |
| 94 | (EuroQual* adj3 5* Dimension*).mp. | 8 |
| 95 | (EuroQual* adj5 (index* or instrument* or measur* or questionnaire* or scale* or survey*)).mp. | 17 |
| 96 | "Medical Outcome Short-Form 36".mp. | 27 |
| 97 | "Medical Outcome Study 36 Item Short Form".mp. | 120 |
| 98 | "Medical Outcomes Study 36 item Short Form".mp. | 1275 |
| 99 | "Medical Outcomes Study SF-36".mp. | 121 |
| 100 | "Medical Outcomes Study Short Form 12".mp. | 106 |
| 101 | "MOS 36 Item Short Form Health*".mp. | 189 |
| 102 | "MOS sf 36*".mp. | 331 |
| 103 | "SF 12???".mp. | 5416 |
| 104 | "SF12???".mp. | 550 |
| 105 | SF36*.mp. | 1393 |
| 106 | SF-36*.mp. | 22082 |
| 107 | "Short Form 12*".mp. | 2326 |
| 108 | "Short Form 36".mp. | 10522 |
| 109 | "Short Form Twelve*".mp. | 0 |
| 110 | "Short Form Thirty-Six*".mp. | 1 |
| 111 | or/81-110 [ 3 Selected HRQoL Questionnaires ] | 47494 |
| 112 | 80 and 111 [ Hip or Knee Surgery + 3 Selcted HRQoLs ] | 3741 |
| 113 | exp Aged/ or "Aged, 80 and Over"/ or Frail Elderly/ [ Aged 65 years and older ] | 3447610 |
| 114 | exp Geriatrics/ | 31446 |
| 115 | exp Geriatric Assessment/ | 32176 |
| 116 | Geriatric Psychiatry/ | 2516 |
| 117 | exp Health Services for the Aged/ | 18170 |
| 118 | exp Geriatric Nursing/ | 13851 |
| 119 | Geroscience/ [ MeSH 2022 ] | 118 |
| 120 | Centenarians/ [ MeSH 2022 ] | 96 |
| 121 | Nonagenarians/ [ MeSH 2022 ] | 93 |
| 122 | Octagenarians/ [ MeSH 2022 ] | 0 |
| 123 | Cognitive Aging/ | 1066 |
| 124 | "older than 1##".mp. | 106 |
| 125 | "older than 6#".mp. | 8737 |
| 126 | "older than 7#".mp. | 3838 |
| 127 | "older than 8#".mp. | 1522 |
| 128 | "older than 9#".mp. | 170 |
| 129 | ("over 1##" adj8 year?).mp. | 2907 |
| 130 | ("over 6#" adj8 year?).mp. | 12967 |
| 131 | ("over 7#" adj8 year?).mp. | 5912 |
| 132 | ("over 8#" adj8 year?).mp. | 3193 |
| 133 | ("over 9#" adj8 year?).mp. | 1120 |
| 134 | ((old?? or advance?) adj (age or aging or ageing)).mp. | 95983 |
| 135 | ((old?? or elder?? or senior?) adj (patient? or citizen?? or person? or people or geriatric* or population?)).mp. | 228702 |
| 136 | (aged adj2 "10# years").mp. | 1311 |
| 137 | (aged adj2 "6# years").mp. | 52766 |
| 138 | (aged adj2 "65 years").mp. | 24172 |
| 139 | (aged adj2 "7# years").mp. | 29340 |
| 140 | (aged adj2 "8# years").mp. | 15959 |
| 141 | (aged adj2 "9# years").mp. | 5235 |
| 142 | (ag?ing adj1 research).mp. | 2082 |
| 143 | (elder* adj1 patient?).mp. | 71167 |
| 144 | (old adj age).mp. | 29852 |
| 145 | (old* adj1 patient?).mp. | 87942 |
| 146 | (older adult* or older client* or older patient* or older person* or older people).mp. | 173457 |
| 147 | centenarian*.mp. | 2106 |
| 148 | (cognitive adj2 ag?ing).mp. | 4331 |
| 149 | elder?.mp. | 18411 |
| 150 | elderly.mp. | 265077 |
| 151 | geriatri*.mp. | 111605 |
| 152 | geropsych*.mp. | 475 |
| 153 | geroscien*.mp. | 375 |
| 154 | gerosurg*.mp. | 2 |
| 155 | grandfather*.mp. | 1434 |
| 156 | grandma??.mp. | 137 |
| 157 | grandmother*.mp. | 2822 |
| 158 | grandpa??.mp. | 48 |
| 159 | grandparent*.mp. | 3622 |
| 160 | nonagenarian*.mp. | 1537 |
| 161 | nonagenary.mp. | 0 |
| 162 | octagenarian*.mp. | 46 |
| 163 | octogenarian*.mp. | 3465 |
| 164 | octogenary.mp. | 5 |
| 165 | oncogeriatri*.mp. | 193 |
| 166 | onco-geriatri*.mp. | 42 |
| 167 | orthogeriatri*.mp. | 498 |
| 168 | ortho-geriatri*.mp. | 37 |
| 169 | postmaturity.mp. | 320 |
| 170 | post-maturity.mp. | 99 |
| 171 | psychogeriatri*.mp. | 1768 |
| 172 | psycho-geriatri*.mp. | 125 |
| 173 | retiree*.mp. | 1668 |
| 174 | retirement?.mp. | 21074 |
| 175 | senior citizen*.mp. | 1524 |
| 176 | septuagenarian*.mp. | 406 |
| 177 | septuagenary.mp. | 0 |
| 178 | sexagenarian*.mp. | 98 |
| 179 | sexagenary.mp. | 2 |
| 180 | supercentenarian*.mp. | 117 |
| 181 | super-centenarian*.mp. | 8 |
| 182 | "6# year?".mp. | 323001 |
| 183 | "7# year?".mp. | 215471 |
| 184 | "8# year?".mp. | 125838 |
| 185 | "9# year?".mp. | 53091 |
| 186 | "10# year?".mp. | 35785 |
| 187 | "age? 6#".mp. | 109064 |
| 188 | "age? 7#".mp. | 44002 |
| 189 | "age? 8#".mp. | 1019380 |
| 190 | "age? 9#".mp. | 5719 |
| 191 | "age? 10#".mp. | 4162 |
| 192 | "extreme age?".mp. | 401 |
| 193 | (oldest adj2 old?).mp. | 2833 |
| 194 | (older adj2 adult?).mp. | 103100 |
| 195 | or/113-194 [ Aged or Elderly ] | 3829987 |
| 196 | 112 and 195 [ Hip or Knee Fracture, Injury, Surgery + 3 Selected HRQoLs + Elderly ] | 2502 |
| 197 | remove duplicates from 196 | 2500 |
| 198 | limit 197 to english language | 2404 |

# MEDLINE In-Process

Ovid MEDLINE(R) Epub Ahead of Print and In-Process, In-Data-Review & Other Non-Indexed Citations May 12, 2023

| **#** | **Searches** | **Results** |
| --- | --- | --- |
| 1 | exp knee arthroplasty/ or total knee replacement/ | 0 |
| 2 | (knee? adj3 arthroplast*).mp. | 5739 |
| 3 | (knee? adj3 replac*).mp. | 2054 |
| 4 | exp knee prosthesis/ | 0 |
| 5 | exp knee/ and exp joint prosthesis/ | 0 |
| 6 | (knee? adj3 prosthe*).mp. | 587 |
| 7 | (knee? adj3 implant*).mp. | 327 |
| 8 | Arthroplasty, Replacement, Knee/ | 0 |
| 9 | Knee Prosthesis/ | 0 |
| 10 | ((knee or knees) adj3 replac*).mp. | 2054 |
| 11 | ((knee or knees) adj3 arthroplast*).mp. | 5739 |
| 12 | ((knee or knees) adj3 prosthe*).mp. | 587 |
| 13 | (total adj2 knee?).mp. | 5484 |
| 14 | tka.tw. [Total Knee Arthroplasty] | 2869 |
| 15 | tkr.tw. [Total Knee Replacement] | 428 |
| 16 | tkas.tw. | 443 |
| 17 | tkrs.tw. | 53 |
| 18 | or/1-17 | 7380 |
| 19 | Knee/ | 0 |
| 20 | exp Knee Joint/ | 0 |
| 21 | exp Knee Injuries/ | 0 |
| 22 | Osteoarthritis, Knee/ | 0 |
| 23 | Patella/in [Injuries] | 0 |
| 24 | Patella/su [Surgery] | 0 |
| 25 | knee.mp. | 26600 |
| 26 | knees.mp. | 3570 |
| 27 | or/19-26 | 27628 |
| 28 | exp Arthroplasty/ | 0 |
| 29 | exp Joint Injury/ | 0 |
| 30 | exp Joint Prosthesis/ | 0 |
| 31 | exp Joints/su [Surgery] | 0 |
| 32 | exp Leg/su | 0 |
| 33 | exp Orthopedic Procedures/ | 2 |
| 34 | Osteoporotic Fractures/ | 0 |
| 35 | "Prostheses and Implants"/ | 1 |
| 36 | exp Prosthesis Failure/ | 0 |
| 37 | arthroplast*.mp. | 14423 |
| 38 | hemiarthroplast*.mp. | 801 |
| 39 | art#ficial*.mp. | 50106 |
| 40 | hemi-arthroplast*.mp. | 46 |
| 41 | prosthe*.mp. | 15823 |
| 42 | redo.mp. | 881 |
| 43 | re-do.mp. | 135 |
| 44 | replac*.mp. | 73122 |
| 45 | repair*.mp. | 55309 |
| 46 | revision?.mp. | 17379 |
| 47 | re-revision?.mp. | 115 |
| 48 | rerevision?.mp. | 17 |
| 49 | implant*.mp. | 60453 |
| 50 | endoprosthe*.mp. | 698 |
| 51 | (surgery or surgeries or surgical* or operation? or operative??).tw,kw. | 374481 |
| 52 | or/28-51 | 562389 |
| 53 | 27 and 52 | 14767 |
| 54 | 18 or 53 [Knee Surgery & Related Terms] | 14906 |
| 55 | Arthroplasty, Replacement, Hip/ | 0 |
| 56 | Hip Prosthesis/ | 0 |
| 57 | (exp Hip/ or exp Hip Dislocation/ or exp Hip Dislocation, Congenital/ or exp Hip Fractures/ or exp Hip Injuries/ or exp Hip Joint/ or exp Osteoarthritis, Hip/) and exp joint prosthesis/ | 0 |
| 58 | (exp Hip/ or exp Hip Dislocation/ or exp Hip Dislocation, Congenital/ or exp Hip Fractures/ or exp Hip Injuries/ or exp Hip Joint/ or exp Osteoarthritis, Hip/) and su.fs. | 0 |
| 59 | (exp Femur Head/ or exp Femur Head Necrosis/) and exp joint prosthesis/ | 0 |
| 60 | (exp Femur Head/ or exp Femur Head Necrosis/) and su.fs. | 0 |
| 61 | exp Hip Fractures/ | 0 |
| 62 | acetabular*.mp. | 2654 |
| 63 | ((femur or femoral) adj3 neck*).mp. | 3096 |
| 64 | ((femur or femoral) adj3 fractur*).mp. | 4233 |
| 65 | femoral head prosthe*.mp. | 7 |
| 66 | femur head prosthe*.mp. | 0 |
| 67 | femoroacetabul*.mp. | 886 |
| 68 | (hip? adj3 prosthe*).mp. | 643 |
| 69 | (hip? adj3 arthroplast*).mp. | 5501 |
| 70 | (hip? adj3 replac*).mp. | 2148 |
| 71 | (hip? adj3 implant*).mp. | 375 |
| 72 | intertrochanteric*.mp. | 691 |
| 73 | 52 and (exp Hip/ or exp Hip Dislocation/ or exp Hip Dislocation, Congenital/ or exp Hip Fractures/ or exp Hip Injuries/ or exp Hip Joint/ or exp Osteoarthritis, Hip/) | 0 |
| 74 | (total adj2 hip?).mp. | 5761 |
| 75 | trochanter*.mp. | 1310 |
| 76 | (tha and hip).tw. [Total Hip Arthroplasty] | 2237 |
| 77 | (thr and hip).tw. [Total Hip Replacement] | 343 |
| 78 | (uka and hip).tw. [Unicompartmental Hip Replacement] | 27 |
| 79 | or/55-78 [ Hip Replacement & related terms ] | 15065 |
| 80 | 54 or 79 [ Knee or Hip Fracture, Injury, Surgery ] | 27766 |
| 81 | "12 item Short Form*".mp. | 286 |
| 82 | "12-Item* Short Form*".mp. | 287 |
| 83 | EQ-5D*.mp. | 2003 |
| 84 | EQ5D*.mp. | 171 |
| 85 | EQ5D*.mp. | 171 |
| 86 | EQ5D5*.mp. | 12 |
| 87 | EuroQoL*.mp. | 1256 |
| 88 | EuroQol-5*.mp. | 726 |
| 89 | EuroQol5*.mp. | 5 |
| 90 | (Europ* adj2 (qol or quality of life) adj2 5*).mp. | 119 |
| 91 | (Europ* adj2 (qol or quality of life) adj2 (index* or instrument* or measur* or questionnaire* or scale* or survey*)).mp. | 73 |
| 92 | (European Quality of Life* adj2 Dimension*).mp. | 117 |
| 93 | European Quality of Life* Five* Dimension*.mp. | 18 |
| 94 | (EuroQual* adj3 5* Dimension*).mp. | 1 |
| 95 | (EuroQual* adj5 (index* or instrument* or measur* or questionnaire* or scale* or survey*)).mp. | 3 |
| 96 | "Medical Outcome Short-Form 36".mp. | 1 |
| 97 | "Medical Outcome Study 36 Item Short Form".mp. | 16 |
| 98 | "Medical Outcomes Study 36 item Short Form".mp. | 92 |
| 99 | "Medical Outcomes Study SF-36".mp. | 5 |
| 100 | "Medical Outcomes Study Short Form 12".mp. | 9 |
| 101 | "MOS 36 Item Short Form Health*".mp. | 38 |
| 102 | "MOS sf 36*".mp. | 34 |
| 103 | "SF 12???".mp. | 865 |
| 104 | "SF12???".mp. | 104 |
| 105 | SF36*.mp. | 215 |
| 106 | SF-36*.mp. | 3005 |
| 107 | "Short Form 12*".mp. | 342 |
| 108 | "Short Form 36".mp. | 1312 |
| 109 | "Short Form Twelve*".mp. | 0 |
| 110 | "Short Form Thirty-Six*".mp. | 0 |
| 111 | or/81-110 [ 3 Selected HRQoL Questionnaires ] | 7163 |
| 112 | 80 and 111 [ Hip or Knee Surgery + 3 Selcted HRQoLs ] | 608 |
| 113 | exp Aged/ or "Aged, 80 and Over"/ or Frail Elderly/ [ Aged 65 years and older ] | 0 |
| 114 | exp Geriatrics/ | 0 |
| 115 | exp Geriatric Assessment/ | 0 |
| 116 | Geriatric Psychiatry/ | 0 |
| 117 | exp Health Services for the Aged/ | 0 |
| 118 | exp Geriatric Nursing/ | 0 |
| 119 | Geroscience/ [ MeSH 2022 ] | 0 |
| 120 | Centenarians/ [ MeSH 2022 ] | 0 |
| 121 | Nonagenarians/ [ MeSH 2022 ] | 0 |
| 122 | Octagenarians/ [ MeSH 2022 ] | 0 |
| 123 | Cognitive Aging/ | 0 |
| 124 | "older than 1##".mp. | 12 |
| 125 | "older than 6#".mp. | 1057 |
| 126 | "older than 7#".mp. | 359 |
| 127 | "older than 8#".mp. | 168 |
| 128 | "older than 9#".mp. | 22 |
| 129 | ("over 1##" adj8 year?).mp. | 547 |
| 130 | ("over 6#" adj8 year?).mp. | 2006 |
| 131 | ("over 7#" adj8 year?).mp. | 706 |
| 132 | ("over 8#" adj8 year?).mp. | 424 |
| 133 | ("over 9#" adj8 year?).mp. | 161 |
| 134 | ((old?? or advance?) adj (age or aging or ageing)).mp. | 13964 |
| 135 | ((old?? or elder?? or senior?) adj (patient? or citizen?? or person? or people or geriatric* or population?)).mp. | 36539 |
| 136 | (aged adj2 "10# years").mp. | 158 |
| 137 | (aged adj2 "6# years").mp. | 7541 |
| 138 | (aged adj2 "65 years").mp. | 3627 |
| 139 | (aged adj2 "7# years").mp. | 3547 |
| 140 | (aged adj2 "8# years").mp. | 1984 |
| 141 | (aged adj2 "9# years").mp. | 619 |
| 142 | (ag?ing adj1 research).mp. | 353 |
| 143 | (elder* adj1 patient?).mp. | 11234 |
| 144 | (old adj age).mp. | 3732 |
| 145 | (old* adj1 patient?).mp. | 14422 |
| 146 | (older adult* or older client* or older patient* or older person* or older people).mp. | 28757 |
| 147 | centenarian*.mp. | 326 |
| 148 | (cognitive adj2 ag?ing).mp. | 898 |
| 149 | elder?.mp. | 2327 |
| 150 | elderly.mp. | 37043 |
| 151 | geriatri*.mp. | 7927 |
| 152 | geropsych*.mp. | 45 |
| 153 | geroscien*.mp. | 81 |
| 154 | gerosurg*.mp. | 0 |
| 155 | grandfather*.mp. | 179 |
| 156 | grandma??.mp. | 27 |
| 157 | grandmother*.mp. | 369 |
| 158 | grandpa??.mp. | 14 |
| 159 | grandparent*.mp. | 521 |
| 160 | nonagenarian*.mp. | 218 |
| 161 | nonagenary.mp. | 0 |
| 162 | octagenarian*.mp. | 7 |
| 163 | octogenarian*.mp. | 542 |
| 164 | octogenary.mp. | 0 |
| 165 | oncogeriatri*.mp. | 28 |
| 166 | onco-geriatri*.mp. | 9 |
| 167 | orthogeriatri*.mp. | 152 |
| 168 | ortho-geriatri*.mp. | 5 |
| 169 | postmaturity.mp. | 7 |
| 170 | post-maturity.mp. | 8 |
| 171 | psychogeriatri*.mp. | 120 |
| 172 | psycho-geriatri*.mp. | 8 |
| 173 | retiree*.mp. | 205 |
| 174 | retirement?.mp. | 2442 |
| 175 | senior citizen*.mp. | 206 |
| 176 | septuagenarian*.mp. | 77 |
| 177 | septuagenary.mp. | 0 |
| 178 | sexagenarian*.mp. | 24 |
| 179 | sexagenary.mp. | 2 |
| 180 | supercentenarian*.mp. | 13 |
| 181 | super-centenarian*.mp. | 1 |
| 182 | "6# year?".mp. | 58116 |
| 183 | "7# year?".mp. | 36997 |
| 184 | "8# year?".mp. | 20266 |
| 185 | "9# year?".mp. | 7485 |
| 186 | "10# year?".mp. | 5743 |
| 187 | "age? 6#".mp. | 14168 |
| 188 | "age? 7#".mp. | 5105 |
| 189 | "age? 8#".mp. | 1962 |
| 190 | "age? 9#".mp. | 613 |
| 191 | "age? 10#".mp. | 472 |
| 192 | "extreme age?".mp. | 68 |
| 193 | (oldest adj2 old?).mp. | 404 |
| 194 | (older adj2 adult?).mp. | 18734 |
| 195 | or/113-194 [ Aged or Elderly ] | 190991 |
| 196 | 112 and 195 [ Hip or Knee Fracture, Injury, Surgery + 3 Selected HRQoLs + Elderly ] | 159 |
| 197 | remove duplicates from 196 | 159 |
| 198 | limit 197 to english language | 158 |

# Embase

Embase Classic+Embase 1947 to 2023 May 12

| **#** | **Searches** | **Results** |
| --- | --- | --- |
| 1 | exp knee arthroplasty/ | 41727 |
| 2 | (knee? adj3 arthroplast*).mp,kw. | 52792 |
| 3 | (knee? adj3 replac*).mp,kw. | 35211 |
| 4 | exp knee implant/ | 13504 |
| 5 | exp knee prosthesis/ | 13504 |
| 6 | knee replacement/ | 6698 |
| 7 | exp total knee arthroplasty/ | 18409 |
| 8 | (knee? adj3 art#ficial*).mp,kw. | 435 |
| 9 | (knee? adj3 prosthe*).mp,kw. | 16440 |
| 10 | (knee? adj3 implant*).mp,kw. | 3012 |
| 11 | Arthroplasty, Replacement, Knee/ | 6306 |
| 12 | Knee Prosthesis/ | 10452 |
| 13 | ((knee or knees) adj3 replac*).mp,kw. | 35211 |
| 14 | ((knee or knees) adj3 arthroplast*).mp,kw. | 52791 |
| 15 | ((knee or knees) adj3 prosthe*).mp,kw. | 16440 |
| 16 | total knee replacement/ | 31339 |
| 17 | (total adj2 knee?).mp,kw. | 48328 |
| 18 | tka.mp,kw. [Total Knee Arthroplasty] | 18869 |
| 19 | rtka.mp,kw. [Revised Total Knee Arthroplasty] | 268 |
| 20 | tkr.mp,kw. [Total Knee Replacement] | 4061 |
| 21 | rtkr.mp,kw. [Revised Total Knee Replacement] | 24 |
| 22 | tkas.mp,kw. | 3217 |
| 23 | tkrs.mp,kw. | 574 |
| 24 | patella prosthesis/ [Embase] | 97 |
| 25 | or/1-24 | 73320 |
| 26 | Knee/ | 89558 |
| 27 | exp Knee Joint/ | 90042 |
| 28 | exp Knee Injuries/ | 40607 |
| 29 | Osteoarthritis, Knee/ | 23713 |
| 30 | Patella/ and injur*.mp. | 3157 |
| 31 | Patella/su [Surgery] | 795 |
| 32 | exp knee/ [Embase] | 90042 |
| 33 | exp knee disease/ [Embase] | 110709 |
| 34 | (knee or knees).mp,kw. | 303260 |
| 35 | knee fracture/ | 930 |
| 36 | or/26-35 | 308965 |
| 37 | exp Arthroplasty/ | 107215 |
| 38 | exp Joint Prosthesis/ | 77987 |
| 39 | "Prostheses and Implants"/ | 16239 |
| 40 | prosthesis/ [Embase] | 41432 |
| 41 | prosthesis design/ [Embase] | 7445 |
| 42 | prosthesis dislocation/ [Embase] | 1629 |
| 43 | prosthesis fixation/ [Embase] | 2828 |
| 44 | prosthesis implantation/ [Embase] | 3170 |
| 45 | prosthesis infection/ [Embase] | 7679 |
| 46 | prosthesis loosening/ [Embase] | 12980 |
| 47 | prosthesis material/ [Embase] | 4523 |
| 48 | art#ficial*.mp,kw. | 711209 |
| 49 | arthroplast*.mp,kw. | 132356 |
| 50 | operati*.mp,kw. | 1848490 |
| 51 | periprosthet*.mp,kw. | 16623 |
| 52 | periprosthes*.mp,kw. | 77 |
| 53 | prosthe*.mp,kw. | 376160 |
| 54 | re-do.mp,kw. | 1874 |
| 55 | redo.mp,kw. | 11272 |
| 56 | replac*.mp,kw. | 791531 |
| 57 | repair*.mp,kw. | 623678 |
| 58 | revision?.mp,kw. | 161062 |
| 59 | re-revision?.mp. | 748 |
| 60 | rerevision?.mp. | 207 |
| 61 | hemiarthroplast*.mp,kw. | 6058 |
| 62 | implant*.mp,kw. | 811834 |
| 63 | endoprosthe*.mp,kw. | 24434 |
| 64 | surgery.mp,kw. | 4566167 |
| 65 | surgeries.mp,kw. | 100721 |
| 66 | surgical*.mp,kw. | 2215920 |
| 67 | exp Prosthesis Failure/ | 38880 |
| 68 | or/37-67 | 7965114 |
| 69 | 36 and 68 | 170890 |
| 70 | 25 or 69 [Knee Surgery & Related Terms] | 172156 |
| 71 | Arthroplasty, Replacement, Hip/ | 5614 |
| 72 | Hip Prosthesis/ | 12639 |
| 73 | (exp Hip/ or exp Hip Dislocation/ or exp Hip Dislocation, Congenital/ or exp Hip Fractures/ or exp Hip Injuries/ or exp Hip Joint/ or exp Osteoarthritis, Hip/) and exp joint prosthesis/ | 18546 |
| 74 | (exp Hip/ or exp Hip Dislocation/ or exp Hip Dislocation, Congenital/ or exp Hip Fractures/ or exp Hip Injuries/ or exp Hip Joint/ or exp Osteoarthritis, Hip/) and su.fs. | 47055 |
| 75 | (exp Femur Head/ or exp Femur Head Necrosis/) and exp joint prosthesis/ | 2227 |
| 76 | (exp Femur Head/ or exp Femur Head Necrosis/) and su.fs. | 4270 |
| 77 | (acetabulum adj3 fractur*).mp. | 4392 |
| 78 | (acetabulum adj3 prosth*).mp. | 1501 |
| 79 | (femoral adj3 fractur*).mp. | 27485 |
| 80 | (femur adj3 fractur*).mp. | 47902 |
| 81 | (hip? adj3 arthroplast*).mp. | 50161 |
| 82 | (hip? adj3 arthroscop*).mp. | 5173 |
| 83 | (hip? adj3 implant*).mp. | 3594 |
| 84 | (hip? adj3 prosthe*).mp. | 46787 |
| 85 | (hip? adj3 replac*).mp. | 35145 |
| 86 | (hip? adj3 surg*).mp. | 20082 |
| 87 | (hip? and break*).mp. | 2410 |
| 88 | (hip? and broke*).mp. | 698 |
| 89 | (hip? and fractur*).mp. | 79365 |
| 90 | (trochanter adj2 fractur*).mp. | 279 |
| 91 | (femoral head adj3 prosthe*).mp. | 385 |
| 92 | (femur head adj3 prosthe*).mp. | 1322 |
| 93 | or/71-92 | 207548 |
| 94 | exp Hip/ or exp Hip Dislocation/ or exp Hip Dislocation, Congenital/ or exp Hip Fractures/ or exp Hip Injuries/ or exp Hip Joint/ or exp Osteoarthritis, Hip/ or hip.mp. or hips.mp. | 349193 |
| 95 | "Prostheses and Implants"/ | 16239 |
| 96 | exp Arthroplasty/ | 107215 |
| 97 | exp Arthroscopy/ | 39056 |
| 98 | exp Joint Prosthesis/ | 77987 |
| 99 | exp Postoperative Complications/ | 851231 |
| 100 | exp Prosthesis Failure/ | 38880 |
| 101 | arthroplast*.mp. | 132356 |
| 102 | arthroscop*.mp. | 60843 |
| 103 | art#ficial*.mp,kw. | 711209 |
| 104 | endoprosthe*.mp. | 24434 |
| 105 | fractur*.mp. | 511346 |
| 106 | hemiarthroplast*.mp. | 6058 |
| 107 | implant*.mp. | 811834 |
| 108 | operati*.mp. | 1848490 |
| 109 | prosthe*.mp. | 376160 |
| 110 | replac*.mp. | 791531 |
| 111 | repair*.mp. | 623678 |
| 112 | revision?.mp. | 161062 |
| 113 | re-revision?.mp. | 748 |
| 114 | rerevision?.mp. | 207 |
| 115 | surgery.mp. | 4566167 |
| 116 | surgeries.mp. | 100721 |
| 117 | surgical*.mp. | 2215920 |
| 118 | (tha and hip).tw. [Total Hip Arthroplasty] | 14281 |
| 119 | (thr and hip).tw. [Total Hip Replacement] | 3539 |
| 120 | (uka and hip).tw. [Unicompartmental Hip Replacement ] | 118 |
| 121 | or/95-120 | 8366901 |
| 122 | 94 and (68 or 121) | 226517 |
| 123 | 93 or 122 [ Hip Fracture / Hip Surgery ] | 256588 |
| 124 | hip/ or exp hip arthroplasty/ or hip disarticulation prosthesis/ or hip disease/ or exp hip fracture/ or hip hemiarthroplasty/ or exp hip injury/ or exp hip osteoarthritis/ or hip osteotomy/ or exp hip prosthesis/ or exp hip replacement/ or hip resurfacing device/ or hip stem/ or exp hip surgery/ | 200277 |
| 125 | 123 or 124 [ Hip Fracture, Injury, Surgery ] | 299685 |
| 126 | 70 or 125 [ Knee or Hip Fracture, Injury, Surgery ] | 434644 |
| 127 | exp "European Quality of Life 5 Dimensions Questionnaire"/ | 14272 |
| 128 | "European Quality of Life 5 Dimensions 3 Level Questionnaire"/ | 1863 |
| 129 | "European Quality of Life 5 Dimensions 5 Level Questionnaire"/ | 3758 |
| 130 | Short Form 12/ | 9690 |
| 131 | Short Form 36/ | 39055 |
| 132 | "12 item Short Form*".mp. | 2196 |
| 133 | "12-Item* Short Form*".mp. | 2207 |
| 134 | EQ-5D*.mp. | 23267 |
| 135 | EQ5D*.mp. | 2966 |
| 136 | EQ5D*.mp. | 2966 |
| 137 | EQ5D5*.mp. | 140 |
| 138 | EuroQoL*.mp. | 12440 |
| 139 | EuroQol-5*.mp. | 6494 |
| 140 | EuroQol5*.mp. | 81 |
| 141 | (Europ* adj2 (qol or quality of life) adj2 5*).mp. | 14945 |
| 142 | (Europ* adj2 (qol or quality of life) adj2 (index* or instrument* or measur* or questionnaire* or scale* or survey*)).mp. | 649 |
| 143 | (European Quality of Life* adj2 Dimension*).mp. | 14854 |
| 144 | European Quality of Life* Five* Dimension*.mp. | 156 |
| 145 | (EuroQual* adj3 5* Dimension*).mp. | 19 |
| 146 | (EuroQual* adj5 (index* or instrument* or measur* or questionnaire* or scale* or survey*)).mp. | 38 |
| 147 | "Medical Outcome Short-Form 36".mp. | 34 |
| 148 | "Medical Outcome Study 36 Item Short Form".mp. | 175 |
| 149 | "Medical Outcomes Study 36 item Short Form".mp. | 1640 |
| 150 | "Medical Outcomes Study SF-36".mp. | 186 |
| 151 | "Medical Outcomes Study Short Form 12".mp. | 140 |
| 152 | "MOS 36 Item Short Form Health*".mp. | 294 |
| 153 | "MOS sf 36*".mp. | 563 |
| 154 | "SF 12???".mp. | 10702 |
| 155 | "SF12???".mp. | 1464 |
| 156 | SF36*.mp. | 4329 |
| 157 | SF-36*.mp. | 42717 |
| 158 | "Short Form 12*".mp. | 10759 |
| 159 | "Short Form 36".mp. | 43570 |
| 160 | "Short Form Twelve*".mp. | 0 |
| 161 | "Short Form Thirty-Six*".mp. | 4 |
| 162 | or/127-161 [ Three Selected HRQoL Questionnaires ] | 105229 |
| 163 | 126 and 162 [ Knee or Hip + 3 HRQoLs ] | 7516 |
| 164 | "Aged, 80 and Over"/ | 211371 |
| 165 | Frail Elderly/ | 12098 |
| 166 | exp Geriatrics/ | 50070 |
| 167 | exp Gerontology/ | 4457 |
| 168 | Geriatric Psychiatry/ | 8076 |
| 169 | exp Health Services for the Aged/ | 82026 |
| 170 | exp Geriatric Nursing/ | 13188 |
| 171 | Geroscience/ | 3129 |
| 172 | Centenarians/ | 286694 |
| 173 | Nonagenarians/ | 272269 |
| 174 | Octogenarians/ | 286694 |
| 175 | "older than 1##".ti,ab. | 171 |
| 176 | "older than 6#".ti,ab. | 15776 |
| 177 | "older than 7#".ti,ab. | 7082 |
| 178 | "older than 8#".ti,ab. | 2935 |
| 179 | "older than 9#".ti,ab. | 317 |
| 180 | ("over 1##" adj8 year?).ti,ab. | 4849 |
| 181 | ("over 6#" adj8 year?).ti,ab. | 23920 |
| 182 | ("over 7#" adj8 year?).ti,ab. | 11088 |
| 183 | ("over 8#" adj8 year?).ti,ab. | 6246 |
| 184 | ("over 9#" adj8 year?).ti,ab. | 2168 |
| 185 | ((old?? or advance?) adj (age or aging or ageing)).ti,ab. | 167607 |
| 186 | ((old?? or elder?? or senior?) adj (patient? or citizen?? or person? or people or geriatric* or population?)).ti,ab. | 392913 |
| 187 | (aged adj2 "10# years").ti,ab. | 2147 |
| 188 | (aged adj2 "6# years").ti,ab. | 82056 |
| 189 | (aged adj2 "65 years").ti,ab. | 38596 |
| 190 | (aged adj2 "7# years").ti,ab. | 46764 |
| 191 | (aged adj2 "8# years").ti,ab. | 25634 |
| 192 | (aged adj2 "9# years").ti,ab. | 8201 |
| 193 | (ag?ing adj1 research).ti,ab. | 3010 |
| 194 | (elder* adj1 patient?).ti,ab. | 130237 |
| 195 | (old adj age).ti,ab. | 46124 |
| 196 | (old* adj1 patient?).ti,ab. | 163301 |
| 197 | (older adult* or older client* or older patient* or older person* or older people).ti,ab. | 268444 |
| 198 | centenarian*.ti,ab. | 2936 |
| 199 | elder?.ti,ab. | 28088 |
| 200 | elderly.ti,ab. | 417921 |
| 201 | geriatri*.ti,ab. | 98480 |
| 202 | gerontol*.ti,ab. | 14269 |
| 203 | geropsych*.ti,ab. | 668 |
| 204 | gerosurg*.ti,ab. | 3 |
| 205 | grandfather*.ti,ab. | 3109 |
| 206 | grandma??.ti,ab. | 222 |
| 207 | grandmother*.ti,ab. | 5333 |
| 208 | grandpa??.ti,ab. | 81 |
| 209 | grandparent*.ti,ab. | 5142 |
| 210 | nonagenarian*.ti,ab. | 2535 |
| 211 | nonagenary.ti,ab. | 1 |
| 212 | octagenarian*.ti,ab. | 112 |
| 213 | octogenarian*.ti,ab. | 6478 |
| 214 | octogenary.ti,ab. | 7 |
| 215 | oncogeriatri*.ti,ab. | 324 |
| 216 | onco-geriatri*.ti,ab. | 103 |
| 217 | orthogeriatri*.ti,ab. | 1129 |
| 218 | ortho-geriatri*.ti,ab. | 107 |
| 219 | postmaturity.mp. | 1308 |
| 220 | post-maturity.mp. | 247 |
| 221 | psychogeriatri*.ti,ab. | 4209 |
| 222 | psycho-geriatri*.ti,ab. | 203 |
| 223 | retiree*.ti,ab. | 2286 |
| 224 | retirement?.ti,ab. | 21943 |
| 225 | senior citizen*.ti,ab. | 2261 |
| 226 | septuagenarian*.ti,ab. | 716 |
| 227 | septuagenary.ti,ab. | 0 |
| 228 | sexagenarian*.ti,ab. | 164 |
| 229 | sexagenary.ti,ab. | 1 |
| 230 | supercentenarian*.ti,ab. | 144 |
| 231 | super-centenarian*.ti,ab. | 14 |
| 232 | aged/ or aged hospital patient/ or frail elderly/ or institutionalized elderly/ or very elderly/ [ Embase] | 3765178 |
| 233 | elderly care/ or exp geriatric care/ or home for the aged/ [ Embase] | 81277 |
| 234 | geriatric care/ or geriatric hospital/ or geriatric nursing/ or geriatric patient/ or geriatric surgery/ or geriatrician/ or geriatrics/ [Embase] | 104046 |
| 235 | gerontologic nurse practitioner/ or gerontological research/ or gerontologist/ or gerontology/ [Embase] | 5619 |
| 236 | cognitive aging/ | 2736 |
| 237 | postmaturity/ | 1075 |
| 238 | or/164-237 [ Aged or Elderly ] | 4222788 |
| 239 | 163 and 238 [ (Knee or Hip) + 3 HRQoLs + Elderly ] | 3578 |
| 240 | limit 239 to english language | 3472 |
| 241 | limit 240 to (conference abstracts or "preprints (unpublished, non-peer reviewed)" or (books or chapter or conference abstract or conference paper or "conference review" or "preprint (unpublished, non-peer reviewed)") or (book or book series or conference proceeding or "preprint archive (unpublished, non-peer reviewed)" or trade journal)) | 377 |
| 242 | 240 not 241 [ Conference or non-journal material removed ] | 3095 |
| 243 | 242 not ((exp animals/ or exp animal experimentation/ or nonhuman/) not ((exp animals/ or exp animal experimentation/ or nonhuman/) and exp human/)) | 3095 |
| 244 | limit 242 to human | 3092 |
| 245 | 243 or 244 [ human double check ] | 3095 |
| 246 | remove duplicates from 245 [ Removal of internal database duplicate citations ] | 3032 |
| 247 | 246 [ (Knee or Hip) + 3 HRQoLs + Elderly; limits applied: English, human, conference or non-journal materials removed ] | 3032 |

# CCTR

EBM Reviews - Cochrane Central Register of Controlled Trials April 2023

| **#** | **Searches** | **Results** |
| --- | --- | --- |
| 1 | exp knee arthroplasty/ | 3370 |
| 2 | (knee? adj3 arthroplast*).mp,kw. | 8573 |
| 3 | (knee? adj3 replac*).mp,kw. | 6350 |
| 4 | exp knee implant/ | 0 |
| 5 | exp knee prosthesis/ | 886 |
| 6 | knee replacement/ | 14 |
| 7 | exp total knee arthroplasty/ | 3370 |
| 8 | (knee? adj3 art#ficial*).mp,kw. | 76 |
| 9 | (knee? adj3 prosthe*).mp,kw. | 1537 |
| 10 | (knee? adj3 implant*).mp,kw. | 374 |
| 11 | Arthroplasty, Replacement, Knee/ | 3370 |
| 12 | Knee Prosthesis/ | 886 |
| 13 | ((knee or knees) adj3 replac*).mp,kw. | 6349 |
| 14 | ((knee or knees) adj3 arthroplast*).mp,kw. | 8573 |
| 15 | ((knee or knees) adj3 prosthe*).mp,kw. | 1537 |
| 16 | total knee replacement/ | 3370 |
| 17 | (total adj2 knee?).mp,kw. | 8192 |
| 18 | tka.mp,kw. [Total Knee Arthroplasty] | 3564 |
| 19 | rtka.mp,kw. [Revised Total Knee Arthroplasty] | 13 |
| 20 | tkr.mp,kw. [Total Knee Replacement] | 770 |
| 21 | rtkr.mp,kw. [Revised Total Knee Replacement] | 1 |
| 22 | tkas.mp,kw. | 292 |
| 23 | tkrs.mp,kw. | 48 |
| 24 | patella prosthesis/ [Embase] | 1 |
| 25 | or/1-24 | 10660 |
| 26 | Knee/ | 1588 |
| 27 | exp Knee Joint/ | 4290 |
| 28 | exp Knee Injuries/ | 1643 |
| 29 | Osteoarthritis, Knee/ | 5932 |
| 30 | Patella/ and injur*.mp. | 105 |
| 31 | Patella/su [Surgery] | 0 |
| 32 | exp knee/ [Embase] | 1588 |
| 33 | exp knee disease/ [Embase] | 0 |
| 34 | (knee or knees).mp,kw. | 37993 |
| 35 | knee fracture/ | 2 |
| 36 | or/26-35 | 38197 |
| 37 | exp Arthroplasty/ | 6859 |
| 38 | exp Joint Prosthesis/ | 2237 |
| 39 | "Prostheses and Implants"/ | 728 |
| 40 | prosthesis/ [Embase] | 49 |
| 41 | prosthesis design/ [Embase] | 2867 |
| 42 | prosthesis dislocation/ [Embase] | 2 |
| 43 | prosthesis fixation/ [Embase] | 5 |
| 44 | prosthesis implantation/ [Embase] | 578 |
| 45 | prosthesis infection/ [Embase] | 4 |
| 46 | prosthesis loosening/ [Embase] | 12 |
| 47 | prosthesis material/ [Embase] | 8 |
| 48 | art#ficial*.mp,kw. | 26119 |
| 49 | arthroplast*.mp,kw. | 14895 |
| 50 | operati*.mp,kw. | 111778 |
| 51 | periprosthet*.mp,kw. | 577 |
| 52 | periprosthes*.mp,kw. | 1 |
| 53 | prosthe*.mp,kw. | 16896 |
| 54 | re-do.mp,kw. | 82 |
| 55 | redo.mp,kw. | 416 |
| 56 | replac*.mp,kw. | 47744 |
| 57 | repair*.mp,kw. | 19009 |
| 58 | revision?.mp,kw. | 4756 |
| 59 | re-revision?.mp. | 5 |
| 60 | rerevision?.mp. | 0 |
| 61 | hemiarthroplast*.mp,kw. | 531 |
| 62 | implant*.mp,kw. | 43621 |
| 63 | endoprosthe*.mp,kw. | 446 |
| 64 | surgery.mp,kw. | 260917 |
| 65 | surgeries.mp,kw. | 12617 |
| 66 | surgical*.mp,kw. | 123022 |
| 67 | exp Prosthesis Failure/ | 884 |
| 68 | or/37-67 | 412915 |
| 69 | 36 and 68 | 18270 |
| 70 | 25 or 69 [Knee Surgery & Related Terms] | 18466 |
| 71 | Arthroplasty, Replacement, Hip/ | 2356 |
| 72 | Hip Prosthesis/ | 1251 |
| 73 | (exp Hip/ or exp Hip Dislocation/ or exp Hip Dislocation, Congenital/ or exp Hip Fractures/ or exp Hip Injuries/ or exp Hip Joint/ or exp Osteoarthritis, Hip/) and exp joint prosthesis/ | 509 |
| 74 | (exp Hip/ or exp Hip Dislocation/ or exp Hip Dislocation, Congenital/ or exp Hip Fractures/ or exp Hip Injuries/ or exp Hip Joint/ or exp Osteoarthritis, Hip/) and su.fs. | 1929 |
| 75 | (exp Femur Head/ or exp Femur Head Necrosis/) and exp joint prosthesis/ | 60 |
| 76 | (exp Femur Head/ or exp Femur Head Necrosis/) and su.fs. | 155 |
| 77 | (acetabulum adj3 fractur*).mp. | 114 |
| 78 | (acetabulum adj3 prosth*).mp. | 106 |
| 79 | (femoral adj3 fractur*).mp. | 2593 |
| 80 | (femur adj3 fractur*).mp. | 1899 |
| 81 | (hip? adj3 arthroplast*).mp. | 5546 |
| 82 | (hip? adj3 arthroscop*).mp. | 309 |
| 83 | (hip? adj3 implant*).mp. | 239 |
| 84 | (hip? adj3 prosthe*).mp. | 2417 |
| 85 | (hip? adj3 replac*).mp. | 5043 |
| 86 | (hip? adj3 surg*).mp. | 3500 |
| 87 | (hip? and break*).mp. | 487 |
| 88 | (hip? and broke*).mp. | 53 |
| 89 | (hip? and fractur*).mp. | 7066 |
| 90 | (trochanter adj2 fractur*).mp. | 15 |
| 91 | (femoral head adj3 prosthe*).mp. | 17 |
| 92 | (femur head adj3 prosthe*).mp. | 45 |
| 93 | or/71-92 | 16769 |
| 94 | exp Hip/ or exp Hip Dislocation/ or exp Hip Dislocation, Congenital/ or exp Hip Fractures/ or exp Hip Injuries/ or exp Hip Joint/ or exp Osteoarthritis, Hip/ or hip.mp. or hips.mp. | 28694 |
| 95 | "Prostheses and Implants"/ | 728 |
| 96 | exp Arthroplasty/ | 6859 |
| 97 | exp Arthroscopy/ | 1953 |
| 98 | exp Joint Prosthesis/ | 2237 |
| 99 | exp Postoperative Complications/ | 48623 |
| 100 | exp Prosthesis Failure/ | 884 |
| 101 | arthroplast*.mp. | 14895 |
| 102 | arthroscop*.mp. | 5778 |
| 103 | art#ficial*.mp,kw. | 26119 |
| 104 | endoprosthe*.mp. | 446 |
| 105 | fractur*.mp. | 27526 |
| 106 | hemiarthroplast*.mp. | 531 |
| 107 | implant*.mp. | 43621 |
| 108 | operati*.mp. | 111778 |
| 109 | prosthe*.mp. | 16896 |
| 110 | replac*.mp. | 47744 |
| 111 | repair*.mp. | 19009 |
| 112 | revision?.mp. | 4756 |
| 113 | re-revision?.mp. | 5 |
| 114 | rerevision?.mp. | 0 |
| 115 | surgery.mp. | 260917 |
| 116 | surgeries.mp. | 12617 |
| 117 | surgical*.mp. | 123022 |
| 118 | (tha and hip).tw. [Total Hip Arthroplasty] | 1513 |
| 119 | (thr and hip).tw. [Total Hip Replacement] | 450 |
| 120 | (uka and hip).tw. [Unicompartmental Hip Replacement ] | 9 |
| 121 | or/95-120 | 432046 |
| 122 | 94 and (68 or 121) | 17125 |
| 123 | 93 or 122 [ Hip Fracture / Hip Surgery ] | 19004 |
| 124 | hip/ or exp hip arthroplasty/ or hip disarticulation prosthesis/ or hip disease/ or exp hip fracture/ or hip hemiarthroplasty/ or exp hip injury/ or exp hip osteoarthritis/ or hip osteotomy/ or exp hip prosthesis/ or exp hip replacement/ or hip resurfacing device/ or hip stem/ or exp hip surgery/ | 6751 |
| 125 | 123 or 124 [ Hip Fracture, Injury, Surgery ] | 20005 |
| 126 | 70 or 125 [ Knee or Hip Fracture, Injury, Surgery ] | 35228 |
| 127 | exp "European Quality of Life 5 Dimensions Questionnaire"/ | 0 |
| 128 | "European Quality of Life 5 Dimensions 3 Level Questionnaire"/ | 24 |
| 129 | "European Quality of Life 5 Dimensions 5 Level Questionnaire"/ | 62 |
| 130 | Short Form 12/ | 126 |
| 131 | Short Form 36/ | 744 |
| 132 | "12 item Short Form*".mp. | 492 |
| 133 | "12-Item* Short Form*".mp. | 496 |
| 134 | EQ-5D*.mp. | 9328 |
| 135 | EQ5D*.mp. | 1394 |
| 136 | EQ5D*.mp. | 1394 |
| 137 | EQ5D5*.mp. | 80 |
| 138 | EuroQoL*.mp. | 4791 |
| 139 | EuroQol-5*.mp. | 2424 |
| 140 | EuroQol5*.mp. | 39 |
| 141 | (Europ* adj2 (qol or quality of life) adj2 5*).mp. | 3197 |
| 142 | (Europ* adj2 (qol or quality of life) adj2 (index* or instrument* or measur* or questionnaire* or scale* or survey*)).mp. | 639 |
| 143 | (European Quality of Life* adj2 Dimension*).mp. | 3170 |
| 144 | European Quality of Life* Five* Dimension*.mp. | 79 |
| 145 | (EuroQual* adj3 5* Dimension*).mp. | 14 |
| 146 | (EuroQual* adj5 (index* or instrument* or measur* or questionnaire* or scale* or survey*)).mp. | 29 |
| 147 | "Medical Outcome Short-Form 36".mp. | 11 |
| 148 | "Medical Outcome Study 36 Item Short Form".mp. | 25 |
| 149 | "Medical Outcomes Study 36 item Short Form".mp. | 451 |
| 150 | "Medical Outcomes Study SF-36".mp. | 31 |
| 151 | "Medical Outcomes Study Short Form 12".mp. | 24 |
| 152 | "MOS 36 Item Short Form Health*".mp. | 99 |
| 153 | "MOS sf 36*".mp. | 110 |
| 154 | "SF 12???".mp. | 2776 |
| 155 | "SF12???".mp. | 416 |
| 156 | SF36*.mp. | 1484 |
| 157 | SF-36*.mp. | 13229 |
| 158 | "Short Form 12*".mp. | 1680 |
| 159 | "Short Form 36".mp. | 8469 |
| 160 | "Short Form Twelve*".mp. | 0 |
| 161 | "Short Form Thirty-Six*".mp. | 1 |
| 162 | or/127-161 [ Three Selected HRQoL Questionnaires ] | 32527 |
| 163 | 126 and 162 [ Knee or Hip + 3 HRQoLs ] | 1919 |
| 164 | "Aged, 80 and Over"/ | 62145 |
| 165 | Frail Elderly/ | 997 |
| 166 | exp Geriatrics/ | 398 |
| 167 | exp Gerontology/ | 398 |
| 168 | Geriatric Psychiatry/ | 50 |
| 169 | exp Health Services for the Aged/ | 534 |
| 170 | exp Geriatric Nursing/ | 201 |
| 171 | Geroscience/ | 2 |
| 172 | Centenarians/ | 0 |
| 173 | Nonagenarians/ | 0 |
| 174 | Octogenarians/ | 1 |
| 175 | "older than 1##".ti,ab. | 12 |
| 176 | "older than 6#".ti,ab. | 1157 |
| 177 | "older than 7#".ti,ab. | 480 |
| 178 | "older than 8#".ti,ab. | 141 |
| 179 | "older than 9#".ti,ab. | 8 |
| 180 | ("over 1##" adj8 year?).ti,ab. | 170 |
| 181 | ("over 6#" adj8 year?).ti,ab. | 2149 |
| 182 | ("over 7#" adj8 year?).ti,ab. | 721 |
| 183 | ("over 8#" adj8 year?).ti,ab. | 246 |
| 184 | ("over 9#" adj8 year?).ti,ab. | 89 |
| 185 | ((old?? or advance?) adj (age or aging or ageing)).ti,ab. | 5568 |
| 186 | ((old?? or elder?? or senior?) adj (patient? or citizen?? or person? or people or geriatric* or population?)).ti,ab. | 30585 |
| 187 | (aged adj2 "10# years").ti,ab. | 149 |
| 188 | (aged adj2 "6# years").ti,ab. | 13205 |
| 189 | (aged adj2 "65 years").ti,ab. | 7044 |
| 190 | (aged adj2 "7# years").ti,ab. | 8556 |
| 191 | (aged adj2 "8# years").ti,ab. | 3876 |
| 192 | (aged adj2 "9# years").ti,ab. | 589 |
| 193 | (ag?ing adj1 research).ti,ab. | 73 |
| 194 | (elder* adj1 patient?).ti,ab. | 13467 |
| 195 | (old adj age).ti,ab. | 1317 |
| 196 | (old* adj1 patient?).ti,ab. | 9732 |
| 197 | (older adult* or older client* or older patient* or older person* or older people).ti,ab. | 26603 |
| 198 | centenarian*.ti,ab. | 16 |
| 199 | elder?.ti,ab. | 2164 |
| 200 | elderly.ti,ab. | 50649 |
| 201 | geriatri*.ti,ab. | 7178 |
| 202 | gerontol*.ti,ab. | 413 |
| 203 | geropsych*.ti,ab. | 13 |
| 204 | gerosurg*.ti,ab. | 0 |
| 205 | grandfather*.ti,ab. | 21 |
| 206 | grandma??.ti,ab. | 6 |
| 207 | grandmother*.ti,ab. | 112 |
| 208 | grandpa??.ti,ab. | 2 |
| 209 | grandparent*.ti,ab. | 170 |
| 210 | nonagenarian*.ti,ab. | 35 |
| 211 | nonagenary.ti,ab. | 0 |
| 212 | octagenarian*.ti,ab. | 1 |
| 213 | octogenarian*.ti,ab. | 128 |
| 214 | octogenary.ti,ab. | 0 |
| 215 | oncogeriatri*.ti,ab. | 18 |
| 216 | onco-geriatri*.ti,ab. | 7 |
| 217 | orthogeriatri*.ti,ab. | 56 |
| 218 | ortho-geriatri*.ti,ab. | 7 |
| 219 | postmaturity.mp. | 20 |
| 220 | post-maturity.mp. | 14 |
| 221 | psychogeriatri*.ti,ab. | 207 |
| 222 | psycho-geriatri*.ti,ab. | 21 |
| 223 | retiree*.ti,ab. | 61 |
| 224 | retirement?.ti,ab. | 522 |
| 225 | senior citizen*.ti,ab. | 165 |
| 226 | septuagenarian*.ti,ab. | 19 |
| 227 | septuagenary.ti,ab. | 0 |
| 228 | sexagenarian*.ti,ab. | 3 |
| 229 | sexagenary.ti,ab. | 0 |
| 230 | supercentenarian*.ti,ab. | 0 |
| 231 | super-centenarian*.ti,ab. | 0 |
| 232 | aged/ or aged hospital patient/ or frail elderly/ or institutionalized elderly/ or very elderly/ [ Embase] | 253754 |
| 233 | elderly care/ or exp geriatric care/ or home for the aged/ [ Embase] | 83 |
| 234 | geriatric care/ or geriatric hospital/ or geriatric nursing/ or geriatric patient/ or geriatric surgery/ or geriatrician/ or geriatrics/ [Embase] | 761 |
| 235 | gerontologic nurse practitioner/ or gerontological research/ or gerontologist/ or gerontology/ [Embase] | 43 |
| 236 | cognitive aging/ | 59 |
| 237 | postmaturity/ | 1 |
| 238 | or/164-237 [ Aged or Elderly ] | 327933 |
| 239 | 163 and 238 [ (Knee or Hip) + 3 HRQoLs + Elderly ] | 733 |
| 240 | limit 239 to english language | 719 |
| 241 | 240 not (abstract or addresses or bibliography or biography or book or book article or book book or book note or "book review" or book series article or book series article in press or book series chapter or book series conference paper or book series letter or "book series review" or book series short survey or chapter or conference or conference abstract or conference abstract placebo controlled partly blinded crossover study in 12 sle patients or conference proceeding or "conference review" or journal conference abstract or "journal conference review" or monograph or conferenc* or book*).pt. | 700 |
| 242 | 241 not (trial* or protocol*).pt. | 520 |
| 243 | 241 not 242 [ double check of empty trial records ] | 180 |
| 244 | remove duplicates from 242 [ Removal of internal database duplicate citations ] | 513 |
| 245 | 244 [ (Knee or Hip) + 3 HRQoLs + Elderly; limited to English, conference or non-journal material removed ] | 513 |

# CDSR

EBM Reviews - Cochrane Database of Systematic Reviews 2005 to May 9, 2023

| **#** | **Searches** | **Results** |
| --- | --- | --- |
| 1 | (knee? adj3 arthroplast*).ti,ab. | 18 |
| 2 | (knee? adj3 replac*).ti,ab. | 23 |
| 3 | (knee? adj3 prosthe*).ti,ab. | 5 |
| 4 | (knee? adj3 implant*).ti,ab. | 0 |
| 5 | ((knee or knees) adj3 replac*).ti,ab. | 23 |
| 6 | ((knee or knees) adj3 arthroplast*).ti,ab. | 18 |
| 7 | ((knee or knees) adj3 prosthe*).ti,ab. | 5 |
| 8 | (total adj2 knee?).ti,ab. | 22 |
| 9 | tka.tw. [Total Knee Arthroplasty] | 27 |
| 10 | tkr.tw. [Total Knee Replacement] | 22 |
| 11 | tkas.tw. | 5 |
| 12 | tkrs.tw. | 4 |
| 13 | or/1-12 | 52 |
| 14 | knee.ti,ab. | 152 |
| 15 | knees.ti,ab. | 13 |
| 16 | arthroplast*.ti,ab. | 49 |
| 17 | hemiarthroplast*.ti,ab. | 9 |
| 18 | art#ficial*.ti,ab. | 72 |
| 19 | hemi-arthroplast*.ti,ab. | 0 |
| 20 | prosthe*.ti,ab. | 46 |
| 21 | redo.ti,ab. | 0 |
| 22 | re-do.ti,ab. | 0 |
| 23 | replac*.ti,ab. | 365 |
| 24 | repair*.ti,ab. | 159 |
| 25 | revision?.ti,ab. | 33 |
| 26 | re-revision?.ti,ab. | 0 |
| 27 | rerevision?.ti,ab. | 0 |
| 28 | implant*.ti,ab. | 179 |
| 29 | endoprosthe*.ti,ab. | 3 |
| 30 | (surgery or surgeries or surgical* or operation? or operative??).tw,kw. | 5511 |
| 31 | or/16-30 | 5719 |
| 32 | (14 or 15) and 31 | 128 |
| 33 | 13 or 32 [ Knee ] | 136 |
| 34 | acetabular*.ti,ab. | 5 |
| 35 | ((femur or femoral) adj3 neck*).ti,ab. | 8 |
| 36 | ((femur or femoral) adj3 fractur*).ti,ab. | 16 |
| 37 | femoral head prosthe*.ti,ab. | 0 |
| 38 | femur head prosthe*.ti,ab. | 0 |
| 39 | femoroacetabul*.ti,ab. | 1 |
| 40 | (hip? adj3 prosthe*).ti,ab. | 2 |
| 41 | (hip? adj3 arthroplast*).ti,ab. | 23 |
| 42 | (hip? adj3 replac*).ti,ab. | 23 |
| 43 | (hip? adj3 implant*).ti,ab. | 5 |
| 44 | intertrochanteric*.ti,ab. | 0 |
| 45 | (total adj2 hip?).ti,ab. | 28 |
| 46 | trochanter*.ti,ab. | 6 |
| 47 | (tha and hip).tw. [Total Hip Arthroplasty] | 20 |
| 48 | (thr and hip).tw. [Total Hip Replacement] | 19 |
| 49 | (uka and hip).tw. [Unicompartmental knee Replacement] | 3 |
| 50 | or/34-49 [ Hip ] | 75 |
| 51 | 33 or 50 [ Knee or Hip ] | 189 |
| 52 | "12 item Short Form*".ti,ab. | 10 |
| 53 | "12-Item* Short Form*".ti,ab. | 10 |
| 54 | EQ-5D*.ti,ab. | 12 |
| 55 | EQ5D*.ti,ab. | 3 |
| 56 | EQ5D*.ti,ab. | 3 |
| 57 | EQ5D5*.ti,ab. | 0 |
| 58 | EuroQoL*.ti,ab. | 11 |
| 59 | EuroQol-5*.ti,ab. | 6 |
| 60 | EuroQol5*.ti,ab. | 0 |
| 61 | (Europ* adj2 (qol or quality of life) adj2 5*).ti,ab. | 1 |
| 62 | (Europ* adj2 (qol or quality of life) adj2 (index* or instrument* or measur* or questionnaire* or scale* or survey*)).ti,ab. | 1 |
| 63 | (European Quality of Life* adj2 Dimension*).ti,ab. | 1 |
| 64 | European Quality of Life* Five* Dimension*.ti,ab. | 0 |
| 65 | (EuroQual* adj3 5* Dimension*).ti,ab. | 0 |
| 66 | (EuroQual* adj5 (index* or instrument* or measur* or questionnaire* or scale* or survey*)).ti,ab. | 0 |
| 67 | "Medical Outcome Short-Form 36".ti,ab. | 0 |
| 68 | "Medical Outcome Study 36 Item Short Form".ti,ab. | 0 |
| 69 | "Medical Outcomes Study 36 item Short Form".ti,ab. | 1 |
| 70 | "Medical Outcomes Study SF-36".ti,ab. | 0 |
| 71 | "Medical Outcomes Study Short Form 12".ti,ab. | 0 |
| 72 | "MOS 36 Item Short Form Health*".ti,ab. | 0 |
| 73 | "MOS sf 36*".ti,ab. | 0 |
| 74 | "SF 12???".ti,ab. | 14 |
| 75 | "SF12???".ti,ab. | 0 |
| 76 | SF36*.ti,ab. | 3 |
| 77 | SF-36*.ti,ab. | 53 |
| 78 | "Short Form 12*".ti,ab. | 2 |
| 79 | "Short Form 36".ti,ab. | 13 |
| 80 | "Short Form Twelve*".ti,ab. | 0 |
| 81 | "Short Form Thirty-Six*".ti,ab. | 0 |
| 82 | or/52-81 [ 3 Selected HRQoL Questionnaires ] | 94 |
| 83 | 51 and 82 [ (Knee or Hip) + 3 HRQoLs ] | 10 |
| 84 | limit 83 to full systematic reviews | 8 |

# Scopus Citing Searching with Topics Added

**Topic components added to citing searching:**

(knee OR knees OR hip OR hips OR trochanter OR femur OR femoral OR acetabular*)

AND

(arthroplast* OR prosthe* or repair* or revision OR rerevision OR implant* or endoprosthe* OR re-do OR redo OR THA OR THR OR TKA OR TKR OR UKA OR surg*)

# 3,152 documents have cited:

Refined to: ( ( ( knee  OR  knees  OR  hip  OR  hips  OR  trochanter  OR  femur  OR  femoral  OR  acetabular* ) ) )  AND  ( ( arthroplast*  OR  prosthe*  OR  repair*  OR  revision  OR  rerevision  OR  implant*  OR  endoprosthe*  OR  re-do  OR  redo  OR  tha  OR  thr  OR  tka  OR  tkr  OR  uka  OR  surg* ) )  AND  ( LIMIT-TO ( DOCTYPE ,  "ar" )  OR  LIMIT-TO ( DOCTYPE ,  "re" )  OR  LIMIT-TO ( DOCTYPE ,  "ed" )  OR  LIMIT-TO ( DOCTYPE ,  "sh" ) )  AND  ( LIMIT-TO ( LANGUAGE ,  "English" ) )

[The MOS 36-item short-form health survey (Sf-36): I. conceptual framework and item selection](https://www-scopus-com.myaccess.library.utoronto.ca/record/display.uri?eid=2-s2.0-0026877917&origin=resultslist&sort=plf-f&cite=2-s2.0-0026877917&src=s&nlo=&nlr=&nls=&imp=t&sid=28d93be28e183fb4043580e46771f414&sot=cite&sdt=cl&cluster=scosubtype%2c%22ar%22%2ct%2c%22re%22%2ct%2c%22ed%22%2ct%2c%22sh%22%2ct%2bscolang%2c%22English%22%2ct&sl=0&ref=%28%28%28knee+OR+knees+OR+hip+OR+hips+OR+trochanter+OR+femur+OR+femoral+OR+acetabular*%29%29%29+AND+%28%28arthroplast*+OR+prosthe*+or+repair*+or+revision+OR+rerevision+OR+implant*+or+endoprosthe*+OR+re-do+OR+redo+OR+THA+OR+THR+OR+TKA+OR+TKR+OR+UKA+OR+surg*%29%29)

[Ware J.E.](https://www-scopus-com.myaccess.library.utoronto.ca/authid/detail.uri?origin=resultslist&authorId=7201752048&zone=), [Sherbourne C.D.](https://www-scopus-com.myaccess.library.utoronto.ca/authid/detail.uri?origin=resultslist&authorId=57204297954&zone=)

(1992) Medical Care, 30 (6) , pp. 473-483.

# 1,520 documents have cited:

Refined to: ( ( ( knee  OR  knees  OR  hip  OR  hips  OR  trochanter  OR  femur  OR  femoral  OR  acetabular* ) ) )  AND  ( ( arthroplast*  OR  prosthe*  OR  repair*  OR  revision  OR  rerevision  OR  implant*  OR  endoprosthe*  OR  re-do  OR  redo  OR  tha  OR  thr  OR  tka  OR  tkr  OR  uka  OR  surg* ) )  AND  ( LIMIT-TO ( DOCTYPE ,  "ar" )  OR  LIMIT-TO ( DOCTYPE ,  "re" )  OR  LIMIT-TO ( DOCTYPE ,  "ed" )  OR  LIMIT-TO ( DOCTYPE ,  "tb" )  OR  LIMIT-TO ( DOCTYPE ,  "le" ) )  AND  ( LIMIT-TO ( LANGUAGE ,  "English" ) )

[A 12-Item Short-Form Health Survey: Construction of Scales and Preliminary Tests of Reliability and Validity](https://www-scopus-com.myaccess.library.utoronto.ca/record/display.uri?eid=2-s2.0-0030096228&origin=resultslist&sort=plf-f&cite=2-s2.0-0030096228&src=s&nlo=&nlr=&nls=&imp=t&sid=5eb3c4df3279dd61a1a2c171cf66534c&sot=cite&sdt=cl&cluster=scosubtype%2c%22ar%22%2ct%2c%22re%22%2ct%2c%22ed%22%2ct%2c%22tb%22%2ct%2c%22le%22%2ct%2bscolang%2c%22English%22%2ct&sl=0&ref=%28%28%28knee+OR+knees+OR+hip+OR+hips+OR+trochanter+OR+femur+OR+femoral+OR+acetabular*%29%29%29+AND+%28%28arthroplast*+OR+prosthe*+or+repair*+or+revision+OR+rerevision+OR+implant*+or+endoprosthe*+OR+re-do+OR+redo+OR+THA+OR+THR+OR+TKA+OR+TKR+OR+UKA+OR+surg*%29%29)

[Ware Jr. J.E.](https://www-scopus-com.myaccess.library.utoronto.ca/authid/detail.uri?origin=resultslist&authorId=7201752048&zone=), [Kosinski M.](https://www-scopus-com.myaccess.library.utoronto.ca/authid/detail.uri?origin=resultslist&authorId=35551981500&zone=), [Keller S.D.](https://www-scopus-com.myaccess.library.utoronto.ca/authid/detail.uri?origin=resultslist&authorId=7202555617&zone=)

(1996) Medical Care, 34 (3) , pp. 220-233.

# 1,442 documents have cited:

Refined to: ( ( ( knee  OR  knees  OR  hip  OR  hips  OR  trochanter  OR  femur  OR  femoral  OR  acetabular* ) ) )  AND  ( ( arthroplast*  OR  prosthe*  OR  repair*  OR  revision  OR  rerevision  OR  implant*  OR  endoprosthe*  OR  re-do  OR  redo  OR  tha  OR  thr  OR  tka  OR  tkr  OR  uka  OR  surg* ) )  AND  ( LIMIT-TO ( DOCTYPE ,  "ar" )  OR  LIMIT-TO ( DOCTYPE ,  "re" )  OR  LIMIT-TO ( DOCTYPE ,  "ed" )  OR  LIMIT-TO ( DOCTYPE ,  "tb" ) )  AND  ( LIMIT-TO ( LANGUAGE ,  "English" ) )

[EuroQol - a new facility for the measurement of health-related quality of life](https://www-scopus-com.myaccess.library.utoronto.ca/record/display.uri?eid=2-s2.0-0025688231&origin=resultslist&sort=plf-f&cite=2-s2.0-0025688231&src=s&nlo=&nlr=&nls=&imp=t&sid=68b05a2411d52a67b5f4e6a72da466a2&sot=cite&sdt=cl&cluster=scosubtype%2c%22ar%22%2ct%2c%22re%22%2ct%2c%22ed%22%2ct%2c%22tb%22%2ct%2bscolang%2c%22English%22%2ct&sl=0&ref=%28%28%28knee+OR+knees+OR+hip+OR+hips+OR+trochanter+OR+femur+OR+femoral+OR+acetabular*%29%29%29+AND+%28%28arthroplast*+OR+prosthe*+or+repair*+or+revision+OR+rerevision+OR+implant*+or+endoprosthe*+OR+re-do+OR+redo+OR+THA+OR+THR+OR+TKA+OR+TKR+OR+UKA+OR+surg*%29%29)

(1990) Health policy, 16 (3) , pp. 199-208.

**Scopus Citation Search**

TITLE ( euroqol AND - AND a AND new AND facility AND for AND the AND measurement AND of AND health-related AND quality AND of AND life )

1 results

( TITLE ( a AND 12-item AND short-form AND health AND survey: AND construction AND of AND scales AND preliminary AND tests AND of AND reliability AND validity. ) AND AUTHOR-NAME ( ware AND j* ) ) AND PUBYEAR = 1996

1 results

( TITLE ( the AND mos AND 36-item AND short-form AND health AND survey AND sf-36 AND : AND i. AND conceptual AND framework AND item AND selection

[1 results](https://www-scopus-com.myaccess.library.utoronto.ca/search/history/results.uri?origin=searchhistory&shid=1)

**Three Selected Surveys are:**

Ware, J. E., & Sherbourne, C. D. (1992). **The MOS 36-Item Short-Form Health Survey (SF-36): I. Conceptual Framework and Item Selection**. Medical Care, 30(6), 473–483. PMID: 1593914. <http://www.jstor.org/stable/3765916>

Ware, J. E., Kosinski, M., & Keller, S. D. (1996). **A 12-Item Short-Form Health Survey: Construction of Scales and Preliminary Tests of Reliability and Validity.** Medical Care, 34(3), 220–233. PMID: 8628042. <http://www.jstor.org/stable/3766749>

(1990). **EuroQol - a new facility for the measurement of health-related quality of life.** Health Policy, 16 (3), 199-208. PMID: 10109801.

<https://doi.org/10.1016/0168-8510(90)90421-9>.

The 23 members of the EuroQol Group are listed in the Appendix.

Here are the articles for the HRQoL surveys:

- [**SF- 36**](https://urldefense.com/v3/__https:/www.jstor.org/stable/3765916?casa_token=8w5HMpJ8cJ8AAAAA:6ms6ThkXGQHQ_Bev5R2Qawzu8POl73kZM6sFLVV8d4d2vvjyh8HRuXYZwg6ZA4U2Ojs0quQ0fZBYNnNmcBGuBPFDJw3Dn7q5XsEvfE5DZtnHOLJHDSZ2__;!!CjcC7IQ!JhgpM2pEWqRSVGhD3vo3EV7e2iTkKso-Z9dRg7vwk8Uo4gyuYazQiflBJV4fw7bV97nP3SrhQh0BRJeTvcgn4VLO6CA$)
- [**SF-12**](https://urldefense.com/v3/__https:/www.jstor.org/stable/3766749?casa_token=7ZATDazYwTwAAAAA*3An26twqJG7Gw2xnWOD675NeX1A5-a7zX0aPU4irAiChl6p77g1BnOu65DqPXQG8NnnvUsDEcHL5CNzdhnwAWgzTWxQafBOQIi6fjPWt3tF2BqVAAf2ACe__;JQ!!CjcC7IQ!JhgpM2pEWqRSVGhD3vo3EV7e2iTkKso-Z9dRg7vwk8Uo4gyuYazQiflBJV4fw7bV97nP3SrhQh0BRJeTvcgn1E_b5oc$)
- [**EQ-5D (EuroQol)**](https://urldefense.com/v3/__https:/www.sciencedirect.com/science/article/abs/pii/0168851090904219?via*3Dihub__;JQ!!CjcC7IQ!JhgpM2pEWqRSVGhD3vo3EV7e2iTkKso-Z9dRg7vwk8Uo4gyuYazQiflBJV4fw7bV97nP3SrhQh0BRJeTvcgnkg3yuRY$)
